# Liposomal Mineral Absorption: A Randomized Crossover Trial

**DOI:** 10.3390/nu14163321

**Published:** 2022-08-13

**Authors:** Grant M. Tinsley, Patrick S. Harty, Matthew T. Stratton, Madelin R. Siedler, Christian Rodriguez

**Affiliations:** Energy Balance & Body Composition Laboratory, Department of Kinesiology & Sport Management, Texas Tech University, Lubbock, TX 79409, USA

**Keywords:** iron, magnesium, multivitamin, liposomes, micronutrients, bioavailability, absorption

## Abstract

Multivitamin/mineral (MVM) supplements are one of the most popular dietary supplement categories. The purpose of this analysis was to determine if a novel liposomal delivery mechanism improves mineral absorption from an MVM product. In a randomized crossover trial, 25 healthy participants (12 females, 13 males) completed two testing sessions in which blood samples were collected at baseline and 2, 4, and 6 h following the ingestion of either a liposomal MVM or a nutrient-matched standard MVM. Analysis of MVM products indicated an elemental iron content of 9.4 and 10.1 mg (~50% U.S. FDA Daily Value) and an elemental magnesium content of 22.0 and 23.3 mg (~5% U.S. FDA Daily Value) in the liposomal and standard MVM products, respectively. Blood samples were analyzed for concentrations of iron and magnesium using colorimetric assays. Changes in mineral concentrations were analyzed using linear mixed models, and pharmacokinetic parameters were compared between conditions. For iron, statistically significant condition × time interactions were observed for percent change from baseline (*p* = 0.002), rank of percent change from baseline (*p* = 0.01), and raw concentrations (*p* = 0.02). Follow-up testing indicated that the liposomal condition exhibited larger changes from baseline than the standard MVM condition at 4 (*p* = 0.0001; +14.3 ± 18.5% vs. −6.0 ± 13.1%) and 6 h (*p* = 0.0002; +1.0 ± 20.9% vs. −21.0 ± 15.3%) following MVM ingestion. These changes were further supported by a 50% greater mean incremental area under the curve in the liposomal condition (33.2 ± 30.9 vs. 19.8 ± 19.8 mcg/dL × 6 h; *p* = 0.02, Cohen’s *d* effect size = 0.52). In contrast, no differential effects for magnesium absorption were observed. In conclusion, iron absorption from an MVM product is enhanced by a liposomal delivery mechanism.

## 1. Introduction

Multivitamin/mineral (MVM) supplements have been consumed since the early 1940s and remain popular today [1,2]. National Health and Nutrition Examination Survey (NHANES) data indicate that MVM products are consumed more frequently than any other type of dietary supplement, and that the proportion of the population consuming MVM products increases with age. Based on NHANES 2017–2018 data, it was estimated that MVM products are consumed by 24% of adults aged 20–39 years, 30% of adults aged 40–59 years, and 39% of adults aged 60 years and older [3]. It is also estimated that, in the United States, 14% of all dietary supplement purchases and 38% of all vitamin and mineral sales are attributable to MVM products. 

Several investigations have supported the utility of MVM products for increasing nutrient intake and improving nutrient status [4,5,6,7,8]. However, it is noteworthy that a standardized definition of MVM is not currently available, and a variety of classifications have been used in research, monitoring, and commercial contexts [1,9]. Additionally, distinct subcategories of MVM, such as basic (broad spectrum), high potency, and specialized (condition specific) have been described [1]. These considerations, as well as the multitude of specific formulations used in extant research, preclude clear conclusions regarding the utility of MVM supplements as a broad category [1,10]. As such, the attributes of specific, commercially available MVM products should be evaluated. One relevant consideration is the bioavailability and absorption profiles of individual nutrients contained within a product, factors which are affected by the product characteristics (e.g., formulation, excipients, fillers, coatings, etc.) and dissolution ability [9]. The need to establish accurate composition and bioavailability data across micronutrient products, including MVM, has been noted as a priority for future research [10]. One major challenge to establishing the bioavailability of MVM products is variance between label claims and actual contents. In this regard, production and examination of a certificate of analysis (COA) for each investigated product is warranted to aid in objective verification of nutrient content. Furthermore, it has been noted that minimal bioavailability data are available for MVM products, in contrast to individual nutrient supplements or foods, and it cannot be assumed that the same nutrients within an MVM will be absorbed similarly to a single-ingredient preparation [10]. The unique matrix of nutrients, corresponding nutrient–nutrient interactions, and the physical form of the product (i.e., capsule, tablet, liquid, etc.) may influence the bioavailability of nutrients within an MVM, but limited information is available in this regard [10].

**Figure 1:**
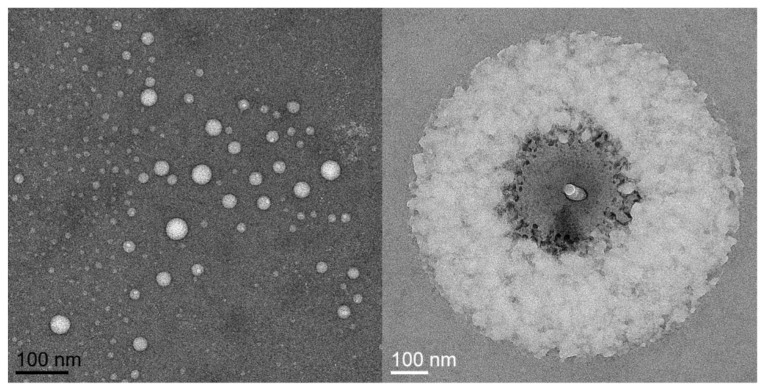
Liposomes as contained in the liposomal multivitamin/mineral product in the present investigation. Images were captured using cryogenic electron microscopy. Images courtesy of Dr. David Belnap, EM Core Lab, University of Utah.

A particularly notable development influencing the physical form of products and potentially altering the bioavailability of nutrients within MVMs is the introduction of liposomal delivery mechanisms, in which nutrients are packaged in liposomes to promote enhanced absorption and bioavailability [11]. Liposomes are spherical vesicles composed of one or more phospholipid bilayers (Figure 1) [12]. This structure allows for packaging of both water- and fat-soluble compounds. Hydrophobic compounds are encapsulated within the interior of the sphere, adjacent to the hydrophilic phospholipid heads, whereas hydrophobic compounds can be accommodated among the hydrophobic fatty acid tails [11]. In some cases, liposomal packaging allows for protection of the contents from a hostile gastrointestinal environment, and also provides the potential for improved cellular uptake due to interactions between the liposomal membrane and the cell membrane. Liposomes have several additional advantages as a compound delivery mode, including increased stability, biocompatibility, and flexibility as compared to conventional methods [12,13]. 

Several investigations support the ability of liposomal packaging to improve vitamin absorption [14,15,16,17]. However, few studies have examined the influence of liposomal packaging on mineral absorption, particularly within the context of an MVM product. This is notable due to well-documented impacts of inadequate intakes of specific minerals, including iron, magnesium, and others [18,19]. The World Health Organization (WHO) estimated that one in three non-pregnant women, corresponding to ~500 million individuals, were anemic in 2011, and that iron deficiency likely contributed to at least half of these cases [20]. Improved understanding of optimal supplementation strategies may help inform interventions to combat these global concerns. In this regard, previous studies have reported the absorption characteristics of non-liposomal forms of iron and magnesium. For example, increases in serum and plasma iron concentrations 2 h following ingestion of iron as iron sulfate or ferroglycin sulphate have been reported [21,22], and the change in iron concentration 2 h following ingestion has been posited as an appropriate time interval for low-dose oral iron absorption tests [22]. Additionally, multiple investigations have reported increased serum magnesium concentrations 4 h after ingestion of supplements containing magnesium citrate or magnesium malate [23,24]. While these investigations do not provide information regarding absorption of minerals from liposomal products, they indicate the potential to examine differential absorption of iron and magnesium several hours after ingestion.

Due to the promise of liposomal technology for compound delivery, additional research is needed to clarify the impact of this delivery mechanism on the pharmacokinetic properties of individual nutrients, such as iron, contained within MVM products. Accordingly, the purpose of the present investigation was to determine if a novel liposomal delivery mechanism improves absorption of iron and magnesium contained in an MVM product. It was hypothesized that superior nutrient absorption would be observed with the liposomal MVM as compared to a nutrient-matched standard MVM product.

## 2. Materials and Methods

### 2.1. Overview

This study was a randomized crossover trial examining the pharmacokinetic profiles of mineral absorption from traditional and liposomal MVM formulations in healthy adults. Each participant completed two research visits, which were identical except for which MVM product was consumed. At each visit, participants reported to the laboratory after an overnight fast. After a baseline blood sample was collected, an MVM product was consumed alongside a standardized breakfast. At 2, 4, and 6 h post-ingestion, additional blood samples were collected. Concentrations of iron and magnesium were quantified, and the pharmacokinetic profiles of each nutrient were examined. The study was conducted according to the guidelines of the Declaration of Helsinki and approved by the Institutional Review Board of Texas Tech University (protocol code 2021-527; date of approval: 22 July 2021). The study was registered on clinicaltrials.gov (identifier: NCT05060367; first posted: 29 September 2021). While the study was originally designed to additionally examine vitamin concentrations, analytical complications at the partner laboratory prevented use of these data.

### 2.2. Participants

Healthy adult participants were recruited for participation. Inclusion criteria were age of 18 to 65 years, body mass of ≥50 kg (due to blood draws), and anticipated ability to comply with study procedures and scheduling requirements. Exclusion criteria were presence of a disease or medical condition—such as cardiovascular disease, cancer, respiratory disease, gastrointestinal disease, or metabolic disease—or current use of medication that could reasonably influence study outcomes or make participation inadvisable; inability to abstain from medication, supplement, or substance ingestion during the overnight fast and duration of the visit; anticipated inability to provide blood samples; current pregnancy or breastfeeding; and allergy that would prevent safe consumption of the standardized breakfast or MVM products. Written informed consent was obtained from all subjects involved in the study. Participants were asked to follow their normal lifestyle practices—including their typical diet—throughout the entire study involvement, with the exception of the pre-visit restrictions described below. At the first testing visit, body composition was estimated using multi-frequency bioelectrical impedance analysis (Seca mBCA 515/514, Seca, Hamburg, Germany). Participant characteristics are displayed in Table 1.

### 2.3. Testing Visits

For each visit, participants reported to the laboratory after an overnight fast (≥12 h) from food, dietary supplements, medications, and intake of all substances except water. Additionally, participants were asked to abstain from any dietary supplement consumption for the three days prior to each testing visit. A baseline blood draw was collected using standard phlebotomy procedures. Blood was collected into serum separation tubes (SST; BD Vacutainer). Processing procedures were based on manufacturer recommendations. Upon collection, the SST were gently inverted five times, then allowed to clot for 30 min in the upright position. Thereafter, the SST were centrifuged at room temperature (22 °C) at a speed of 1000 RCF (g) for 10 min in a swinging bucket centrifuge. After centrifugation, serum samples were aliquoted into microcentrifuge tubes and frozen at −80 °C until shipment to the partner laboratory for analysis. 

Following the baseline blood draw, participants were provided with the standardized breakfast and MVM product. The standardized breakfast consisted of two packages (4 total bars) of Nature Valley Oats n’ Honey Crunchy granola bars. Based on product labeling, this breakfast provided 380 kcal, 14 g fat, 58 g carbohydrate, 6 g protein, 280 mg sodium, and 2 mg iron. Ingredients in this product were whole grain oats, sugar, canola and/or sunflower oil, rice flour, honey, salt, brown sugar syrup, baking soda, soy lecithin, and natural flavor. This product was selected due to its relative lack of micronutrient fortification, unlike most packaged breakfast products. The first package (i.e., two bars) of the breakfast product was consumed, followed by MVM ingestion (liposomal or standard) and consumption of the second package. In a double-blind fashion, each participant ingested one serving (2 capsules) of the specified MVM product. The order of MVM ingestion for each participant was randomized using the *randomizR* software package [25] for R (date of randomization: 8 September 2021). Both MVM products were manufactured by Nutraceutical International Corporation using CELLg8^®^ delivery technology, and certificates of analysis (COAs) were provided for the liposomal multivitamin (Lot #: SRC256641; date: 31 August 2021) and the standard multivitamin (Lot #: SRC258623; date: 2 September 2021). Elemental iron content (from ferrous glycinate) of the liposomal and standard MVM was 9.4 and 10.1 mg, respectively (~50% of U.S. FDA Daily Value). Elemental magnesium content (from magnesium glycinate) of the liposomal and standard MVM was 22.0 and 23.3 mg, respectively (~5% of U.S. FDA Daily Value). The full nutritional content of the liposomal and standard MVM products as obtained from the COAs is displayed in Table 2. The exact time of MVM ingestion was noted, and all subsequent blood draws were based on this time point. Bottled water (Purified Drinking Water, Great Value) was provided to all participants and was allowed ad libitum during the first testing visit, with a matched amount of bottled water provided at the second visit. Water consumption during the two visits was (mean ± SD) 1.8 ± 0.9 L and 2.0 ± 1.0 L, respectively.

At 2, 4, and 6 h after MVM ingestion, additional blood samples were collected and processed using the aforementioned procedures. These time intervals were selected for two reasons: (1) based on similarity to previous research examining absorption of iron and magnesium [21,22,23,24], and (2) due to practical constraints related to performing the study in a university laboratory rather than an inpatient facility. Upon completion of the first study visit, each participant began a washout period of at least seven days before returning to the laboratory to complete the second testing visit based on their scheduling availability (mean ± SD duration of washout period: 7.7 ± 2.0 days). Participants were instructed to continue maintaining their usual lifestyle practices, including diet, during the washout period.

### 2.4. Nutrient Analysis

After collection, all samples were shipped on dry ice to a partner laboratory (Heartland Assays, Ames, IA, USA) for analysis. Magnesium and iron were analyzed using colorimetric assay (HM929, Pointe Scientific, Canton, MI and MAK025, Sigma-Aldrich, St. Louis, MO, USA, respectively). All samples were blinded for analysis, and output was provided to the principal investigator for statistical analysis. Complete data were available for magnesium and iron (*n* = 25 participants; *n* = 200 samples). 

### 2.5. Statistical Analysis

Initially, data were examined for extreme outliers (i.e., values above Q3 + 3xIQR or below Q3–3xIQR). No extreme outliers were observed at any time point for raw iron concentrations. One extreme outlier was observed for iron changes from baseline to 6 h, with no extreme outliers identified at other time intervals. No extreme outliers for magnesium concentrations at baseline, 4 h, and 6 h were observed. Two extreme outliers were present for magnesium concentrations at 2 h after MVM ingestion. For changes in magnesium concentrations, five extreme outliers were identified for the interval from baseline to 2 h, four extreme outliers were identified for the interval from baseline to 4 h, and two extreme outliers were identified for the interval from baseline to 6 h. Based on these findings, rank-based tests were performed to allow for preservation of the entire sample size without concerns regarding the distribution of the data. Additionally, non-rank-based tests were performed with and without inclusion of extreme outliers. For completeness, analysis of raw iron and magnesium concentrations are presented in Appendix A, although these analyses were viewed as secondary due to variation in baseline concentrations rendering their comparison less informative than relative changes.

Data were analyzed in R (version 4.1.2; R Foundation for Statistical Computing, Vienna, Austria). Percent changes from baseline concentrations and ranks of percent changes from baseline were visualized using the *ggplot2* [26] package (v. 3.3.5) with within-subject error bars [27,28]. These data were analyzed using linear mixed-effects models (*nlme* package [29], v. 3.1-153) with a random intercept for participant and a first-order autoregressive (AR1) variance-covariance matrix. These models were fit by maximizing the restricted log-likelihood (REML). In all models, the reference groups were the standard MVM for condition, female for sex, and the baseline time point for time. The fixed effects of condition, time, sex, and their interactions were examined, and significant effects were followed up with pairwise comparisons using the *emmeans* [30] package (v. 1.7.2). Multiple comparisons were accounted for using the Benjamini and Hochberg correction [31]. 

The incremental area under the concentration vs. time curve (iAUC) was calculated using the method of Brouns et al. [32]. As the 2, 4, and 6 h time points were specified relative to MVM ingestion, and a mean ± SD of 9.5 ± 1.8 min elapsed between the baseline blood draw and MVM ingestion, values of 0, 2.158, 4.158, and 6.158 h were used for iAUC and other pharmacokinetic calculations. The *PKNCA* [33] package (v. 0.9.5) was used to establish the maximum observed concentration (Cmax) and time of maximum observed concentration (Tmax). These values were calculated for the entire sample, females only, and males only. The iAUC and Cmax values were examined for extreme outliers, as well as for normality of differences between conditions. When no extreme outliers were present and normality of differences was observed (via visual inspection of QQ plots and Shapiro–Wilk tests), data were analyzed using paired samples *t*-tests. When extreme outliers were present and/or probable normality violations were observed, Wilcoxon signed-rank tests were performed. Accordingly, paired-samples *t*-tests were performed for iron iAUC values in the entire sample, males only, and females only; magnesium iAUC values in females only; and iron and magnesium Cmax values in the entire sample and females only. Wilcoxon signed-rank tests were performed for magnesium iAUC values in the entire sample and males only, as well as magnesium and iron Cmax values in males only. These analyses were performed using the *rstatix* [34] package (v. 0.7.0). Associated metrics of effect size (i.e., Cohen’s *d* for paired *t*-tests (d) and Wilcoxon *r* for Wilcoxon signed-rank tests [*r*]) were also calculated. Cohen’s *d* effect sizes can be interpreted as: <0.2 (negligible), 0.2 to <0.5 (small), 0.5 to <0.8 (medium), and >0.8 (large), and Wilcoxon *r* effect sizes can be interpreted as: 0.1 to <0.3 (small), 0.3 to <0.5 (moderate), and ≥0.5 (large) [34]. Due to the nature of the data, all Tmax values were analyzed using Wilcoxon signed-rank tests. Statistical significance was accepted at *p* < 0.05. 

## 3. Results

### 3.1. Mixed Models

#### 3.1.1. Iron

**Figure 2:**
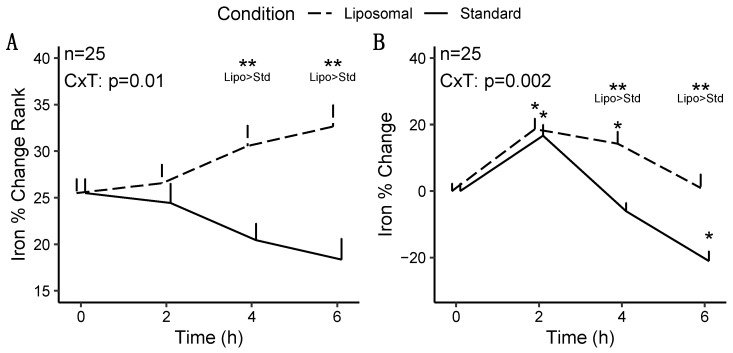
Changes in iron concentrations following multivitamin/mineral ingestion. The rank of percent changes from baseline (**A**) and percent changes from baseline (**B**) are presented. CxT indicates the *p*-value for the condition × time interaction. ** indicates a statistically significant difference between conditions at a particular time point. * indicates a statistically significant difference from baseline concentrations in a particular condition.

For iron, statistically significant condition × time interactions were observed for rank of percent change from baseline (*p* = 0.01; Figure 2A), percent change from baseline (*p* = 0.002; Figure 2B), and raw concentrations (*p* = 0.02; Appendix A). 

Follow-up testing for the significant condition × time interaction for rank of percent change from baseline indicated that the liposomal condition exhibited greater values at 4 (*p* = 0.01) and 6 h (*p* = 0.0003) following MVM ingestion, as compared to the standard MVM condition, without difference at 2 h (*p* = 0.66). A significant time × sex interaction was also present (*p* = 0.006); however, follow-up testing did not reveal any statistically significant pairwise comparisons after correction for multiple comparisons. 

Follow-up testing for the significant condition × time interaction for percent change from baseline indicated that the liposomal condition exhibited greater values than the standard MVM condition at 4 (*p* = 0.0001; +14.3 ± 18.5% vs. −6.0 ± 13.1% [mean ± SD]) and 6 h (*p* = 0.0002; +1.0 ± 20.9% vs. −21.0 ± 15.3%) following MVM ingestion, without difference at 2 h (*p* = 0.84; 18.6 ± 16.2% vs. 16.7 ± 16.9%). A statistically significant condition × time interaction (*p* = 0.002) was also observed in the sensitivity analysis (*n* = 24; removal of one extreme outlier), with statistically significant differences between conditions still observed at the 4 and 6 h time points. Based on the lack of difference in condition effects and interactions between analyses, the results of the full sample are presented. A significant time × sex interaction was also present (*p* = 0.006). Follow-up testing indicated that females presented larger changes in iron at 2 h (*p* = 0.004), as compared to males, but not at other time points (*p* = 0.08 to 1.0). 

#### 3.1.2. Magnesium

**Figure 3:**
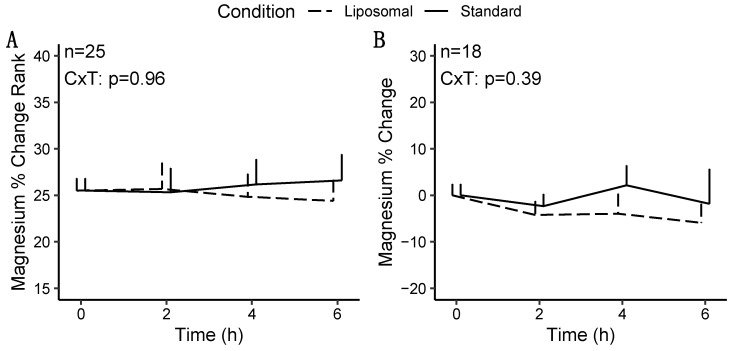
Changes in magnesium concentrations following multivitamin/mineral ingestion. The rank of percent changes from baseline (**A**) and percent changes from baseline after removal of extreme outliers (sensitivity analysis) (**B**) are presented. CxT indicates the *p*-value for the condition × time interaction.

For magnesium, no statistically significant effects of condition, time, or sex were observed in any model (Figure 3), except for a condition × sex × time interaction (*p* = 0.04) for magnesium changes from baseline in the sensitivity analysis (i.e., following removal of extreme outliers; Table 3). However, follow-up testing revealed no significant two-way interactions or pairwise comparisons after correction for multiple comparisons. Based on the difference in statistical significance of the condition × time × sex interaction, the results of the sensitivity analysis are presented.

### 3.2. Pharmacokinetic Analysis

#### 3.2.1. Iron

**Figure 4:**
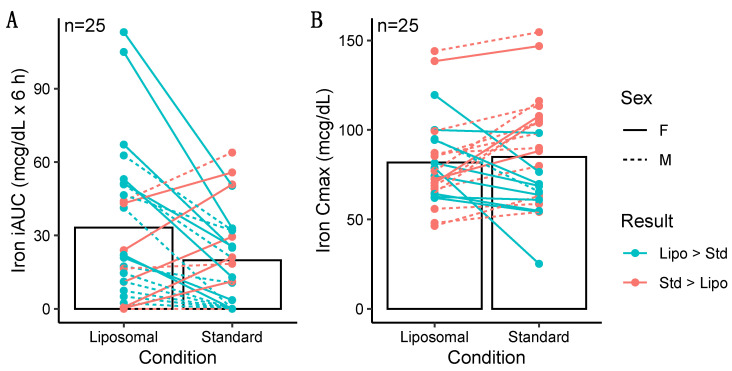
Individual differences in incremental area under the curve (iAUC; panel **A**) and maximal observed concentration (Cmax; panel **B**) values for iron. Iron iAUC values were significantly greater after ingestion of the liposomal product as compared to the standard product (*p* = 0.016 via paired samples *t*-test), with no difference observed for Cmax (*p* = 0.51 via paired samples *t*-test). Individual responses are represented by lines, and bars represent mean values in each condition.

Greater iAUC values were observed for iron in the liposomal MVM condition in the entire sample (*p* = 0.016, *d* = 0.52; Figure 4), with a 50% difference in mean values. Greater iAUC was also observed in males only (*p* = 0.03, *d* = 0.68; Table 4). In females only, the difference in iAUC was not statistically significant (*p* = 0.13, *d* = 0.47), although a similar magnitude of effect size as in the entire sample was observed. 

There was no difference in iron Cmax in the entire sample (*p* = 0.51, *d* = 0.13; Table 5) and females only (*p* = 0.25, *d* = 0.35). In males only, a greater Cmax was observed for iron in the standard MVM condition (*p* = 0.01, *r* = 0.69). However, examination of the data indicated this was due to higher baseline concentrations in the standard MVM condition rather than an increase after ingestion, as further evidenced by the greater iAUC in the liposomal MVM condition. A difference in Tmax for iron was observed in the entire sample (*p* = 0.002, *r* = 0.68; Table 6) and males only (*p* = 0.01, *r* = 0.77), indicating lower (earlier) Tmax values for the standard MVM condition. Tmax values did not significantly differ between conditions in females only (*p* = 0.10, *r* = 0.57). 

#### 3.2.2. Magnesium

**Figure 5:**
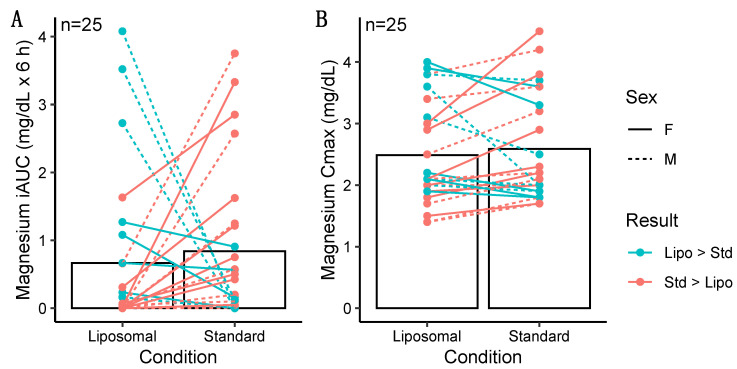
Individual differences in incremental area under the curve (iAUC; panel **A**) and maximal observed concentration (Cmax; panel **B**) values for magnesium. Magnesium iAUC values (*p* = 0.30 via Wilcoxon signed-rank test) and Cmax values (*p* = 0.41 via paired samples *t*-test) did not differ between conditions. Individual responses are represented by lines, and bars represent mean values in each condition.

No differences in magnesium iAUC values were observed in the entire sample (*p* = 0.30, *r* = 0.22; Table 4; Figure 5), males only (*p* = 1.0, *r* = 0.02), or females only (*p* = 0.09, *d* = 0.54). Additionally, no differences between conditions for Cmax values were observed in the entire sample (*p* = 0.41, *d* = 0.17; Table 5), males only (*p* = 0.46, *r* = 0.21), or females only (*p* = 0.27, *d* = 0.34). No differences in Tmax were observed for magnesium in the entire sample (*p* = 0.62, *r* = 0.08; Table 6), males only (*p* = 1.0, *r* = 0.05), or females only (*p* = 0.48, *r* = 0.13).

### 3.3. Side Effects

No side effects related to MVM consumption were reported by participants in either condition. 

## 4. Discussion

The present randomized crossover trial investigated mineral absorption from liposomal and non-liposomal MVM products. As hypothesized, improved iron absorption was observed following ingestion of the liposomal product. Specifically, larger changes in iron from baseline—using both percent changes and ranks of percent changes—were observed in the liposomal condition at 4 and 6 h after MVM ingestion. Additionally, the iAUC for iron was 50% greater following ingestion of the liposomal MVM product. No differences between conditions were observed for magnesium absorption. Importantly, the dose of iron contained in the MVM product represented a relevant dose, whereas the relative dose of magnesium was much lower. The quantity of elemental iron in the MVM products represented ~50% of the 18-mg Daily Value used by the U.S. Food and Drug Administration (FDA) for nutrition labeling purposes [18]. Additionally, the doses of elemental iron used in the present study (9.4 to 10.1 mg) meet the Recommended Dietary Allowance (RDA) for males of all ages, except 14–18 years (RDA: 11 mg/d), and meet or make a substantive contribution to the recommended intake for adult females (RDA: 8 to 18 mg/d, depending on age, in non-pregnant females; 27 mg/d in pregnant females). This indicates that the quantity of iron contained in the MVM product is meaningful relative to daily intake recommendations and supports the relevance of the improved absorption seen with the liposomal formulation. In contrast, the doses of elemental magnesium in the present study were low relative to daily intakes (22 to 23.3 mg vs. U.S. FDA Daily Value of 420 mg; ~5% of Daily Value) [19]. This may indicate that the influence of liposomal delivery on magnesium absorption should be further investigated with higher doses, perhaps through a standalone magnesium supplement due to practical limitations on absolute quantities of ingredients in MVM products. Preliminary research with a form of liposomal magnesium supports this contention, as one study indicated enhanced absorption as compared to non-liposomal forms following ingestion of 350 mg magnesium (i.e., ~15-fold higher than the dose in the present investigation) [35]. This research was conducted within the context of single-nutrient products, in contrast to the present investigation, which examined nutrient absorption from MVM products. 

The observed improvement in iron absorption with liposomal packaging is notable for several reasons. First, iron is essential for a host of physiological functions, ranging from oxygen handling as part of hemoglobin and myoglobin, to hormone synthesis and support of normal cellular function [18]. Second, while previous research has indicated benefits of liposomal delivery for absorption of vitamins [14,15,16,17], there is little information regarding mineral absorption. As such, the present investigation demonstrates the promise of liposomal technology in this regard. Third, the benefit to iron absorption was observed in the context of a relatively bioavailable source of iron, ferrous glycinate. Previous research has demonstrated superior bioavailability of ferrous glycinate as compared to iron salts, such as ferrous sulfate [36,37,38]. As such, it is notable that liposomal packaging further improved absorption. While speculative, it is possible that the enhancement of absorption with liposomal packaging would be even more evident with less bioavailable forms of nutrients. Fourth, the global impact of inadequate iron intake and international recommendations for iron supplementation indicate the importance of using the most effective supplement form. The WHO estimated that one in three non-pregnant women, corresponding to ~500 million individuals, were anemic in 2011, and that iron deficiency likely contributed to at least half of these cases [20]. Correspondingly, a 2016 WHO report recommends daily iron supplementation in menstruating adult women and adolescent girls living in settings where anemia is prevalent [20]. It is well established that groups at risk for inadequate iron intake include adolescent, pregnant, and premenopausal women, as well as infants and children [18]. Racial disparities have also been reported, with higher rates of depleted iron stores in Mexican American and non-Hispanic Black pregnant women [39]. Additionally, those in food-insecure homes are more likely to experience inadequate iron intake [40]. Collectively, these and other lines of evidence indicate the importance of iron supplementation in several contexts and demonstrate the need for effective supplementation formulations.

While few investigations have examined the acute absorption properties of minerals encapsulated in liposomes, some have indicated promise for these products for health improvements following chronic supplementation in clinical conditions [41,42,43,44]. A randomized trial in chronic kidney disease patients demonstrated similar increases in hemoglobin after 3 months of treatment with oral liposomal iron supplements or intravenous iron administration, along with a lower incidence of adverse effects with oral supplementation [41]. A separate single-arm trial in patients with chronic kidney disease indicated that a liposomal iron preparation was well tolerated and increased hemoglobin, relative to baseline, after 12 months of supplementation [42]. In a single-arm trial conducted in anemic patients with inflammatory bowel disease, 62% of patients completing treatment with oral liposomal iron supplements increased hemoglobin above a prespecified threshold or presented with hemoglobin normalization after 8 weeks of treatment; improvements in quality of life and reductions in fatigue were also noted [43]. Finally, a randomized trial in pregnant, non-anemic women indicated that liposomal iron was effective for elevating hemoglobin and ferritin concentrations as compared with control [44]. While these investigations demonstrate the potential utility of liposomal iron formulations, several trials are limited by a lack of control or comparison groups. As such, additional research is needed to investigate the potential for unique health effects in various clinical populations, as well as in the general population. 

While both multi-nutrient (e.g., MVM) and single-nutrient supplements may exhibit benefits in specific contexts, the investigation of individual nutrient absorption from an MVM product is relevant due to the notable prevalence of MVM supplementation. As previously noted, NHANES data indicate that MVM products are consumed more frequently than any other type of dietary supplement [3]. Importantly, the bioavailability of individual nutrients from an MVM product may be dissimilar to absorption from single-nutrient products due to the specific matrix of nutrients and corresponding nutrient–nutrient interactions within an MVM [10]. As such, and due to the dearth of bioavailability data for MVM products, investigations that directly quantity single nutrient absorption from an MVM product—such as the present report—are warranted. 

A major strength of the present investigation is the use of COA to objectively verify the nutrient content of the liposomal and standard MVM products. Interestingly, the benefits of liposomal iron delivery are further highlighted by the fact that the elemental iron content was 7.5% (0.73 mg) lower in the liposomal MVM than the standard MVM, based on the COA. Similarly, the elemental magnesium content was 5.7% lower in the liposomal MVM product. Additional strengths of this study include the rigorous procedural standardization during data collection and multifaceted statistical analysis. Limitations of the present work include the inability to examine additional outcomes; the low dose of magnesium, which limits the relevance of this nutrient as a study outcome; the relatively young, healthy, and homogenous sample; and the limited number of time points utilized. Additionally, the monitoring period of 6 h following MVM ingestion could be a limitation, as a statistically significant difference in iron changes between conditions was present at this time point; therefore, the full duration of differential iron changes could have persisted beyond this point. However, interestingly, iron had returned to near baseline levels in the liposomal condition by 6 h after MVM ingestion, whereas iron concentrations fell below baseline levels in the standard MVM condition. An additional consideration is that while the two MVM products were designed with identical specified amounts of each nutrient, minor differences between the standard and liposomal MVM products were observed through the laboratory COA results. While this may be unavoidable for products with many ingredients and small doses of each individual ingredient, it is worth noting. However, as mentioned, the dose of iron (as well as magnesium) was actually lower in the liposomal MVM, indicating the positive results observed were not due to this slightly different dose. Lastly, the potential influence of compounds within the MVM products on iron and magnesium absorption should be considered. Based on the potential for complex, and largely unknown, interactions between compounds within MVM products [10], the results of the present study could have differed if compounds were studied in isolation.

## 5. Conclusions

In conclusion, the present randomized crossover trial demonstrated improved iron absorption following ingestion of iron from a novel liposomal MVM as compared to a standard MVM. This finding helps to determine optimal iron supplementation strategies and demonstrates the potential for liposomal packaging to benefit mineral absorption. Future research should continue to examine the potential utility of liposomal delivery of micronutrients in a variety of populations, for both MVM formulations and single-nutrient products.

## Figures and Tables

**Table 1:** Participant Characteristics ^1^.

	All (*n* = 25)	Males (*n* = 13)	Females (*n* = 12)
Age (y)	26.0 ± 3.4	26.2 ± 3.6	25.9 ± 3.5
Height (cm)	169.8 ± 9.0	175.9 ± 6.6	163.3 ± 6.1
Weight (kg)	74.7 ± 15.6	83.0 ± 10.9	65.8 ± 15.3
BMI (kg/m^2^)	25.7 ± 3.9	26.8 ± 2.8	24.5 ± 4.6
Body fat (%)	25.4 ± 8.3	21.4 ± 7.2	29.7 ± 7.5

^1^ Values displayed as mean ± SD.

**Table 2:** Nutrient content of multivitamin/mineral products.

Raw Material ^1^	Nutrient	Specified Amount (Unit) ^2^		Std. MVM ^2^	Lipo. MVM ^2^
Vegan Beta Carotene	A	900	mcg	950	1098
Methylcobalamin	B12	1000	mcg	1258	1210
Ascorbic Acid	C	90	mg	108.4	112.7
Vegan Vitamin D-3	D3	20	mcg	22.2	23.9
(6S)-5-Methyltetrahydrofolate	Folic Acid ^3^	235.3	mcg	320.6	326.5
Ferrous Glycinate	Iron *	9	mg	10.1	9.37
Magnesium Glycinate	Magnesium *	20	mg	23.3	22.0
Manganese Citrate	Manganese	2.3	mg	2.6	2.6
Zinc Citrate	Zinc	11	mg	12.8	15.0
Benfotiamine	B1	1.2	mg	1.0	0.9
Riboflavin-5-Phosphate	B2	1.3	mg	0.9	1.1
Niacinamide	B3	16	mg	18.1	17.1
Calcium d-Pantothenate	B5	5	mg	6.4	5.1
Pyridoxine-5-Phospate	B6	1.7	mg	1.0	0.6
Biotin	Biotin	30	mcg	56.8	33.0
Choline Bitartrate	Choline	25	mg	57.6	44.8
Chromium Glycinate	Chromium	35	mcg	54.7	58.0
Co-Q10	Co-Q10	5	mg	4.3	3.8
Sunflower Vitamin E (a-Tocopherol)	E	15	mg	16.4	16.3
Inositol	Inositol	25	mg	19.2	22.3
Potassium Iodide	Iodine	150	mcg	171.9	244.0
K1	K1	120	mcg	129	74.5
Vegan Lutein Beadlet	Lutein	1	mg	1.27	1.25
Molybdenum Glycinate	Molybdenum	45	mcg	54.1	53.6
P-Aminobenzoic Acid	PABA	5	mg	3.4	4.1
Selenium	Selenium	55	mg	54.7	51.8
Acerola CherryJuice Powder	-	2	mg	NT	NT
Trace Minerals	-	5	mg	NT	NT
Rosehips	-	2	mg	NT	NT

Standard MVM and liposomal MVM nutrient content based on certificate of analysis for each product. Nutrient content based on one serving (2 capsules). NT: not tested; ^1^ other ingredients: Veg 00 capsule, cellulose, stearic acid, and silica (manufacturing aids). ^2^ Specified amount and quantities listed for standard and liposomal MVM indicate the quantity of the specified nutrient, not the quantity of raw material. ^3^ Folic Acid is not converted to DFE units; multiply by 1.7 for DFE units. * Study outcome.

**Table 3:** *p*-Values from mixed models.

	Iron			Magnesium		
	Δ Rank	Δ	Δ (S)	Δ Rank	Δ	Δ (S)
*n*	25	25	24	25	25	18
Intercept	<0.001 *	0.72	0.98	<0.001 *	0.13	0.14
Condition	<0.001 *	0.005 *	0.02 *	0.66	0.93	1.00
Time	1.00	<0.001 *	<0.001 *	1.00	0.14	0.12
Sex	0.07	0.045 *	0.08	0.25	0.80	0.77
Condition × Time	0.01 *	0.002 *	0.004 *	0.96	0.79	0.74
Condition × Sex	0.80	0.72	0.64	0.34	0.41	0.44
Time × Sex	0.006 *	0.006 *	0.001 *	0.58	0.36	0.41
Condition × Time × Sex	0.37	0.27	0.29	0.16	0.06	0.04 *

“Δ Rank” indicates rank of percent change from baseline; “Δ” indicates percent change from baseline; “S” indicates sensitivity analysis results (removal of extreme outliers). “*” and bold text indicate statistical significance (i.e., *p* < 0.05).

**Table 4:** Incremental area under the concentration vs. time curve (iAUC) comparison.

	Liposomal MVM				Standard MVM				All		Females		Males	
	Mean	sd	Med	IQR	Mean	sd	Med	IQR	*p*	ES	*p*	ES	*p*	ES
Iron	33.22	30.90	22.00	39.84	19.84	19.75	18.40	32.01	0.02 *	0.52	0.13	0.47	0.03 *	0.68
Mag.	0.67	1.15	0.07	0.67	0.84	1.13	0.43	1.20	0.30	0.22	0.09	0.54	1.00	0.02

Descriptive data for each condition are presented for all participants. Values are in units of incremental area under the time (hours) vs. the concentration curve. Concentration units are mcg/dL for iron and mg/dL for magnesium. *p*-values were generated by a paired samples *t*-test or Wilcoxon signed-rank test, as appropriate (see text for details). Effect sizes (ES) correspond to Cohen’s *d* when paired samples *t*-tests were performed and Wilcoxon *r* when Wilcoxon signed-rank tests were performed. “*” and bold text indicate statistical significance (i.e., *p* < 0.05). sd: standard deviation; med: median; IQR: interquartile range; ES: effect size.

**Table 5:** Maximal observed concentration (Cmax) comparison.

	Liposomal MVM				Standard MVM				All		Females		Males	
	Mean	sd	Med	IQR	Mean	sd	Med	IQR	*p*	ES	*p*	ES	*p*	ES
Iron	81.72	24.72	77.07	29.60	84.94	30.39	79.87	41.89	0.51	0.13	0.25	0.35	0.01 *	0.69
Mag.	2.49	0.84	2.10	1.20	2.59	0.88	2.20	1.40	0.41	0.17	0.27	0.34	0.46	0.21

Descriptive data for each condition are presented for all participants. Units are mcg/dL for iron and mg/dL for magnesium; *p*-values were generated by a paired samples t-test or Wilcoxon signed-rank test, as appropriate (see text for details). Effect sizes (ES) correspond to Cohen’s *d* when paired samples *t*-tests were performed and Wilcoxon *r* when Wilcoxon signed-rank tests were performed. “*” and bold text indicate statistical significance (i.e., *p* < 0.05). sd: standard deviation; med: median; IQR: interquartile range; ES: effect size.

**Table 6:** Time of maximal observed concentration (Tmax) comparison.

	Liposomal MVM				Standard MVM				All		Females		Males	
	Mean	sd	Med	IQR	Mean	sd	Med	IQR	*p*	ES	*p*	ES	*p*	ES
Iron	3.0	1.5	2.2	2.0	1.5	1.0	2.2	2.2	0.002 *	0.68	0.10	0.57	0.01 *	0.77
Mag.	3.2	2.7	4.2	6.2	3.6	2.5	4.2	4.0	0.62	0.08	0.48	0.13	1.00	0.05

Descriptive data for each condition are presented for all participants in units of hours. *p*-values were generated by Wilcoxon signed-rank test, and effect sizes (ES) correspond to Wilcoxon *r*. “*” and bold text indicate statistical significance (i.e., *p* < 0.05). sd: standard deviation; med: median; IQR: interquartile range; ES: effect size.

**Figure A1:**
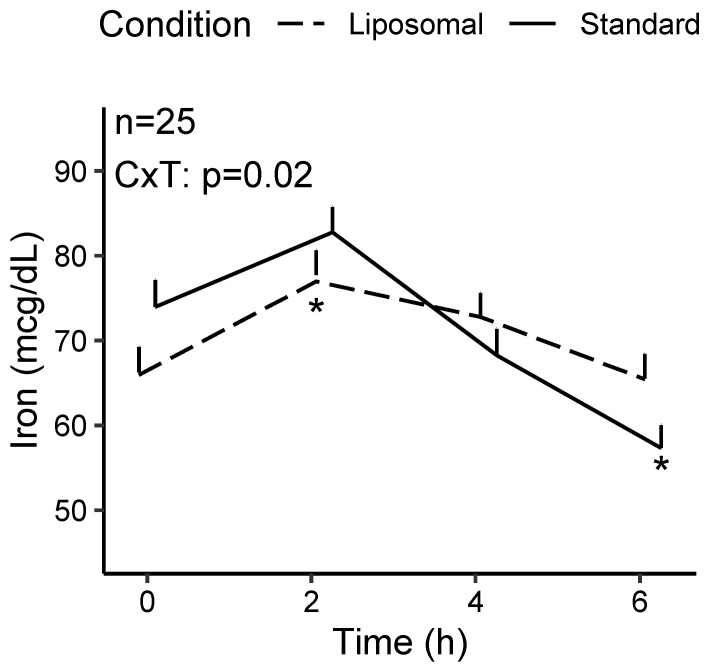
Raw Iron Concentrations. A statistically significant condition × time interaction was observed for raw iron concentrations (*p* = 0.02; Table A1), uncorrected for baseline concentrations. Follow-up testing did not reveal significant differences between conditions at any individual time point (*p* = 0.08 to 0.30). However, iron concentrations in the liposomal MVM condition increased from baseline to 2 h (*p* = 0.03), but were not elevated relative to baseline at any subsequent time point (*p* = 0.14 to *p* = 0.93). In contrast, within the standard MVM condition, the sole significant difference from baseline values was that the final observed value (6 h after MVM ingestion) fell below baseline values (*p* = 0.001), unlike in the liposomal condition (*p* = 0.93). Due to variation in raw baseline concentrations between conditions, the raw changes presented above are viewed as supplemental/secondary, while changes from the baseline and pharmacokinetic parameters (i.e., iAUC) were the primary outcomes. “*” indicate statistical significance (i.e., *p* < 0.05).

**Figure A2:**
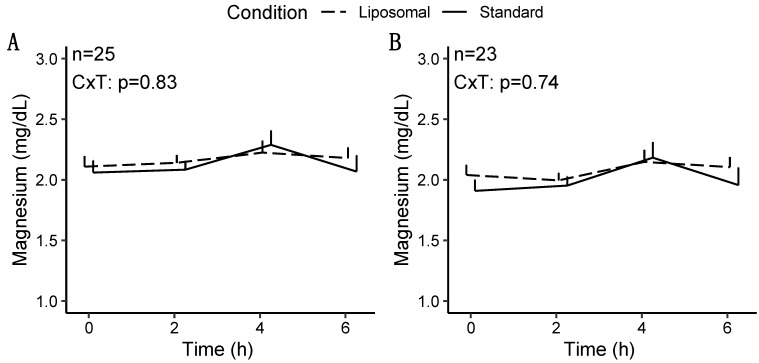
Raw Magnesium Concentrations. No significant condition * time interaction was observed for raw magnesium concentrations, uncorrected for baseline values, in the full sample (**A**) or in a sensitivity analysis with extreme outliers removed (**B**). A significant condition * time * sex interaction was observed in the sensitivity analysis (Table A1); however, follow-up testing did not reveal any statistically significant pairwise comparisons. Due to variation in raw baseline concentrations between conditions, the raw changes presented above are viewed as supplemental/secondary, while changes from the baseline and pharmacokinetic parameters (i.e., iAUC) were the primary outcomes.

## Data Availability

Requests for data will be reviewed by the researchers and associated personnel.

## Appendix A

**Table A1:** *p*-values from mixed models using raw concentrations, uncorrected for baseline values.

	Iron	Magnesium	
	Raw	Raw	Raw (S)
*n*	25	25	23
Intercept	**<0.001 ***	**<0.001 ***	**<0.001 ***
Condition	0.89	0.58	0.34
Time	**<0.001**	0.29	0.14
Sex	0.32	0.88	0.66
Condition × Time	**0.02 ***	0.83	0.74
Condition × Sex	**<0.001 ***	0.38	0.79
Time × Sex	0.40	0.38	0.34
Condition × Time × Sex	0.97	0.09	**0.04 ***

“S” indicates sensitivity analysis results. “*” and bold text indicate statistical significance (i.e., *p* < 0.05).
